# Genome-Wide Identification and Expression Pattern of the *SPP* Gene Family in Cotton (*Gossypium hirsutum*) Under Abiotic Stress

**DOI:** 10.3390/genes16121500

**Published:** 2025-12-15

**Authors:** Cuijie Cui, Chao Wang, Shangfu Ren, Huiqin Wang

**Affiliations:** College of Life and Geography, Kashi University, Kashi 844000, China; ccj19987654321@163.com (C.C.); 13579774188@163.com (C.W.)

**Keywords:** *Gossypium hirsutum*, sucrose phosphate phosphatase, expression profile, gene family, bioinformatics

## Abstract

Background: Sucrose metabolism plays a crucial role in plant responses to abiotic stresses such as drought and high temperatures, significantly influencing plant growth and yield formation. In higher plants, the second step in sucrose bioconversion involves sucrose phosphate phosphatase (SPP) hydrolyzing sucrose-6-phosphate to form sucrose. This study determined the number of *SPP* gene family members in upland cotton (*Gossypium hirsutum*), systematically analyzed their fundamental characteristics, physicochemical properties, phylogenetic relationships, chromosomal localization, and expression patterns across different tissues and under various abiotic stresses. Methods: The *SPP* gene family in *hirsutum* was identified using Hidden Markov Models (HMMER) and the NCBI Conserved Domain Database (NCBI CDD), and its physico-chemical properties were analyzed via the SOPMA online analysis website. Phylogenetic relationships were determined using MEGA 12.0 software. Promoter regions were analyzed with PlantCARE, sequence patterns were identified via MEME, and transcriptome data were downloaded from the CottonMD database. Results: This study identified four members of the *hirsutum SPP* gene family, with amino acid lengths ranging from 335 to 1015, molecular weights between 38.38 and 113.28 kDa, and theoretical isoelectric points (pI) between 5.39 and 6.33. These genes are localized across four chromosomes. The *SPP* gene family in *hirsutum* exhibits closer phylo-genetic relationships with *SPP* genes in *Arabidopsis thaliana* and *Chenopodium quinoa*. Their promoter regions are rich in cis-elements associated with multiple abiotic stress resistance functions, and their expression patterns vary across different tissues and under different abiotic stress conditions. Conclusions: The *GhSPP* gene may play an important role in the growth and development of upland cotton and its responses to salt stress and drought. Therefore, it could be considered as a candidate gene for future functional analysis of cotton resistance to salt and drought stress.

## 1. Introduction

Cotton, as a high-quality natural fiber raw material and important cash crop, holds a significant position in the national economy [1]. China ranks among the world’s largest producers and consumers of raw cotton. With the restructuring of China’s cotton industry, the *Gossypium hirsutum*-growing regions of Northwest China, primarily centered in Xinjiang, have become the core area for cotton production in the country. However, the region faces year-round threats from various natural disasters such as drought, salinity, low temperatures, and high temperatures, making it a typical high-risk cotton-growing area [2,3,4,5]. Therefore, enhancing cotton’s stress tolerance is crucial for ensuring safe cotton production in China.

Sucrose is the primary end product of photosynthesis in higher plants and also serves as the main form for long-distance transport of carbohydrates within plants [6,7]. Sucrose metabolism plays a crucial role in plant responses to abiotic stresses such as drought and high temperatures, significantly influencing plant growth and yield formation [8]. In higher plants, sucrose biosynthesis involves two key steps: In the first step, sucrose phosphate synthase (SPS) catalyzes the conversion of uridine diphosphate glucose and fructose-6-phosphate into sucrose-6-phosphate. In the second step, sucrose phosphate phosphatase (SPP) hydrolyzes sucrose-6-phosphate to form sucrose [9,10]. Despite increasing research on the *SPP* gene, systematic reports on the *SPP* gene family remain scarce. Previous studies have demonstrated that SPP-related family genes can influence the allocation of photosynthetic carbon among different storage compounds [11]. For example, a 2005 study downregulated the expression level of the *SPP* gene in tobacco plants using RNAi technology and found that the content of multiple sugars in the plants decreased significantly [12]. As key enzyme genes in the sucrose synthesis pathway, research on the functions of SPPs in *hirsutum* during its growth and development as well as in responses to abiotic stress remains relatively scarce.

This study comprehensively identified members of the *SPP* gene family across the entire genome of *hirsutum*, systematically analyzing their fundamental characteristics, physicochemical properties, phylogenetic relationships, chromosomal localization, and expression levels in different tissues. These findings provide crucial theoretical foundations for elucidating the biological functions of *SPP* genes in *hirsutum* and the molecular mechanisms regulating sucrose synthesis.

## 2. Materials and Methods

### 2.1. Identification of the SPP Gene Family Members in G. hirsutum

This study downloaded the genome data and annotation files for *hirsutum* from the cotton genome database (https://yanglab.hzau.edu.cn/CottonMD, accessed on 11 December 2025). The identification process for the *SPP* gene family is as follows: First, download the Hidden Markov Model (HMM) files for the S6PP domain (PF05116) and the S6PP_C domain (PF08472) from the Pfam database (http://pfam.xfam.org, accessed on 11 December 2025) [13,14]. The HMMER3 software was utilized to align protein sequences of *hirsutum* against the HMM file, with a threshold of 1 × 10^−5^ for screening the alignment results. Subsequently, these HMM profiles were aligned against the whole-genome protein sequences of *hirsutum* using the HMMER3 software to screen for candidate sequences harboring both the S6PP and S6PP_C domains. Following the initial screening, redundant and incomplete sequences were manually removed. Finally, the obtained candidate protein sequences were submitted to NCBI’s Conserved Domain Database (CDD, https://www.ncbi.nlm.nih.gov/Structure/bwrpsb/bwrpsb.cgi, accessed on 11 December 2025) for domain validation to ensure they indeed contained the complete SPP protein characteristic domains, thereby ultimately confirming the members of the upland cotton *SPP* gene family.

### 2.2. Physicochemical Properties and Secondary Structure Analysis of G. hirsutum SPP Protein

We used the SOPMA online analysis website (https://npsa-prabi.ibcp.fr/cgi-bin/npsa_automat.pl?page=npsa_sopma.html, accessed on 11 December 2025) to analyze the secondary structure (including α-helices, extended chains, β-turns, and random coils) of *hirsutum* SPP protein sequences [15]; The three-dimensional structure analysis of the SPP protein from upland cotton was performed using the online tool Phyre2 (https://www.sbg.bio.ic.ac.uk, accessed on 11 December 2025); the molecular weight, theoretical isoelectric point, and hydrophilicity of the *hirsutum* SPP family proteins were predicted using the ProtParam tool on the ExPASy platform (https://web.expasy.org/protparam/, accessed on 11 December 2025) [16].

### 2.3. Subcellular Localization and Transmembrane Domain Prediction of the SPP Gene Family in G. hirsutum

Use the Plant-mPLoc online tool (http://www.csbio.sjtu.edu.cn/bioinf/plant-multi/, accessed on 11 December 2025) to predict subcellular localization for GhSPP proteins [17]. Employ the TMHMM-2.0 online tool (https://services.healthtech.dtu.dk/, accessed on 11 December 2025) to analyze and predict transmembrane domains in the proteins [18].

### 2.4. Chromosomal Localization and Collinearity Analysis of the G. hirsutum SPP Gene Family

Based on the *hirsutum* genome annotation file (GFF3 format), the TBtools -Ⅱsoftware’s “Gene Location Visualize from GTF/GFF” function was used to determine the specific chromosomal locations of members of the *hirsutum SPP* gene family and generate distribution maps [19]. Subsequently, the built-in “One Step McsanX” tool within TBtools was employed to perform whole-gene synteny analysis [20]. The results were visualized using the “Multiple Synteny Plot” function.

### 2.5. Phylogenetic Analysis of the SPP Gene Family in G. hirsutum

Download SPP protein sequences for *Arabidopsis*, *Zea mays*, *Chenopodium quinoa*, *Oryza sativa* and *Sorghum bicolor* from the UniProt database (https://www.uniprot.org/, accessed on 11 December 2025) [21]. Using MEGA 6.0 software, align the conserved amino acid sequences of the *SPP* gene family members from *hirsutum* with homologous sequences from the aforementioned species. The Neighbor-Joining (NJ) method was employed to construct the phylogenetic tree, with 1000 bootstrap replicates to assess node reliability. Finally, the generated tree was visually enhanced using the Itol online software (https://itol.embl.de/, accessed on 11 December 2025).

### 2.6. Genetic Structure and Analysis of Protein Conservative Motifs in the G. hirsutum SPP Family

Motif analysis of the *hirsutum* SPP gene family was conducted using the MEME software (https://meme-suite.org/meme/, accessed on 11 December 2025). Parameter settings: Maximum motif count set to 6, motif length ranging from 6 to 100 amino acids. Prediction results were visualized using the “MEME Suit Wrapper” function in TBtools software [19].

### 2.7. Promoter Analysis of the G. hirsutum SPP Gene Family

Promoter regions (upstream 2000 bp) of the *SPP* gene family members were extracted from the whole genome sequence of *hirsutum*. The types, quantities, and functions of cis-acting elements were analyzed using the PlantCARE database (https://bioinformatics.psb.ugent.be/webtools/plantcare/html/, accessed on 11 December 2025) [22].

### 2.8. Expression Profile Analysis of the G. hirsutum SPP Gene Family

RNA-seq data obtained from the CottonMD database (https://yanglab.hzau.edu.cn/CottonMD, accessed on 11 December 2025) for different tissues (root, stem, leaf, anther, bract, filament, pistil, sepal, torus, post-flowering fiber at different time points, and ovule) and abiotic stress treatments (low temperature, high temperature, salt, drought). Using FPKM as the expression metric, TBtools software was employed to generate a heatmap of *SPP* gene expression patterns across cotton.

### 2.9. Interaction Network Analysis of Gossypium herbaceum SPP Proteins

To investigate the interaction network of GhSPP proteins in *hirsutum,* this study first constructed the protein interaction network of GhSPP proteins using the online tool from the STRING database (STRING: functional protein association networks). Furthermore, based on the CottonMD database (https://yanglab.hzau.edu.cn/CottonMD, accessed on 11 December 2025), we obtained expression data for the genes encoding proteins interacting with GhSPP in the *hirsutum* standard line TM-1. These data were derived from RNA-seq sequencing results under various abiotic stress treatments (low temperature, high temperature, salt, drought) and normalized using FPKM values to characterize expression levels. Finally, using FPKM as the gene expression metric, TBtools software was employed to generate heatmaps depicting the expression patterns of these interacting protein-encoding genes under multiple stress conditions.

## 3. Results

### 3.1. Identification of the G. hirsutum SPP Gene Family

Using the HMMER software to search the *hirsutum* genome, a total of 4 SPP family members were identified and named *GhSPP1*-*GhSPP4* (Table 1). Analysis of the physicochemical properties of the proteins encoded by these genes indicates that their amino acid lengths range from 335 to 1015, molecular weights fall between 38.38 and 113.28 kDa. Among them, *GhSPP1* has the longest protein length at 1015 amino acids and a molecular weight of 113.28 kDa; *GhSPP2* has the shortest protein length at 335 amino acids and a molecular weight of 38.38 kDa. The theoretical isoelectric points (pI) lie between 5.39 and 6.33. All pI values are below 7, confirming these proteins as acidic. Except for GhSPP2 (with an instability coefficient of 40.81), the instability indices of the other three members are all below 40, classifying them as stable proteins. Additionally, analysis of the hydrophilicity/hydrophobicity of *hirsutum* SPP protein amino acid sequences using the ProtScale tool revealed that all four GhSPP proteins exhibited peak values below −2, containing multiple hydrophilic peaks. Furthermore, the total number of hydrophilic amino acids exceeded that of hydrophobic amino acids. Consequently, it was determined that the SPP protein family in *G. hirsutum* exhibits overall hydrophilic properties.

**Figure 1:**
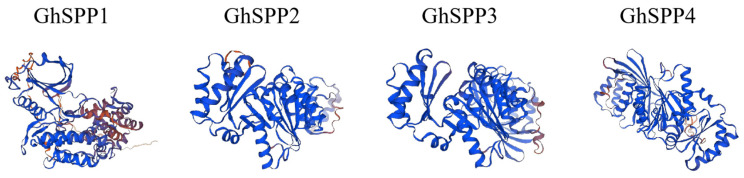
Predicted Three-Dimensional Structure of *G. hirsutum* SPP Protein. The protein structure is presented as a ribbon diagram, with different regions labeled in blue and brown (orange), visually illustrating the distribution of secondary structural elements (such as α-helices and β-sheets) within the GhSPP polypeptide chain and its overall folding characteristics.

Structural modeling and analysis of the GhSPP protein sequence using the Swiss-model and SOPMA online tools (Figure 1, Table 2). The results indicate that all four GhSPP proteins contain fundamental secondary structural elements such as alpha helix, extended strand, random coils, and beta turn, though the proportions of these elements vary significantly among them. From a structural perspective, the GhSPP proteins primarily consist of random coil and alpha helix, which together account for over 70% of its spatial structure. Extended strands constitute the next largest proportion, while beta turn represents the smallest fraction, accounting for less than 10% in all four GhSPP proteins. This indicates that GhSPP proteins exhibit high overall flexibility and dynamicity.

### 3.2. Phylogenetic Analysis of the G. hirsutum SPP Family Genes

**Figure 2:**
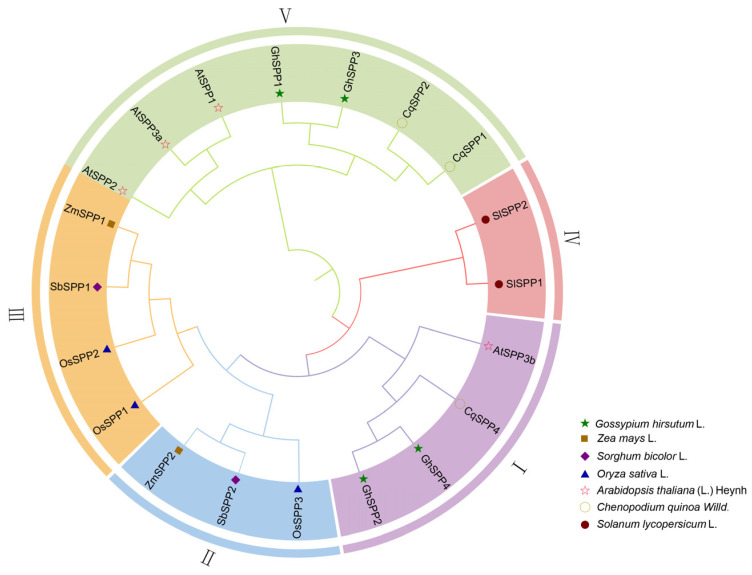
Phylogenetic Tree of the *SPP* Gene Family. Os represents *O. sativa*, Zm represents *Z. mays*, Cq represents *C. quinoa*, Sl represents *Solanum lycopersicum*, Sb represents *S. bicolor*, and At represents *A. thaliana*.

To elucidate the evolutionary origins and phylogenetic relationships of the *SPP* gene family members in *hirsutum*, this study constructed a phylogenetic tree incorporating SPP protein sequences from representative species including *G. hirsutum* (GhSPP), *Z. mays* (ZmSPP), *O. sativa* (OsSPP), *S. bicolor* (SbSPP), *C. quinoa* (CqSPP), and *Arabidopsis thaliana* (AtSPP) (Figure 2). Phylogenetic tree analysis clearly divides all SPP protein sequences into five major evolutionary groups (Group I–Group V). The four *SPP* gene family members of *G. hirsutum* are distributed across two branches: GhSPP2 and GhSPP4 cluster in Group I, showing the closest phylogenetic relationship to CqSPP4 and AtSPP3b; while GhSPP1 and GhSPP3 cluster in Group V, showing closest relationships to AtSPP1, AtSPP2, AtSPP3a, and CqSPP1 and CqSPP2. This finding indicates that the *SPP* gene family in *G. hirsutum* shares closer phylogenetic relationships with corresponding genes in *A. thaliana* and *C. quinoa*, both dicotyledonous plants. This suggests that multiple ancient subfamilies within the *SPP* gene family likely existed prior to the divergence of dicotyledons and monocotyledons, subsequently undergoing independent expansions and evolutions during species-specific evolutionary processes.

### 3.3. Chromosomal Localization and Gene Structure Analysis of the SPP Gene Family in G. hirsutum

**Figure 3:**
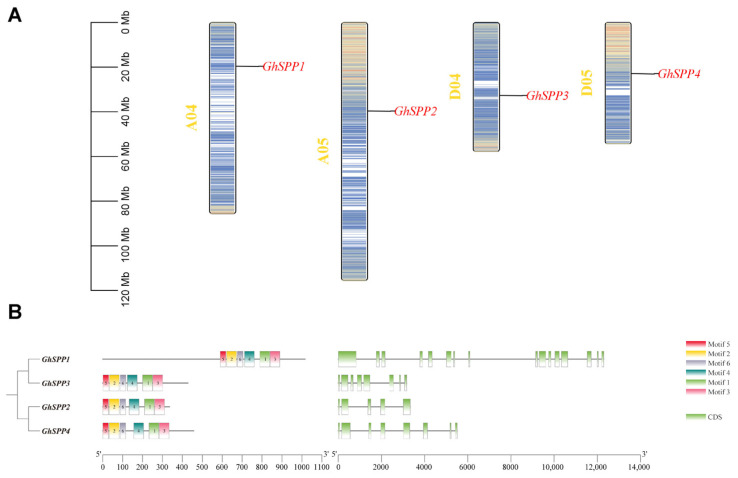
Chromosomal localization and gene structure analysis of the GhSPP Gene Family. (**A**) Chromosome mapping of *SPP* gene family members of *hirsutum.* (**B**) The motif and gene structure of the *SPP* gene family in *hirsutum*.

Using TBtools software to map gene locations on chromosomes (Figure 3A), the four *GhSPP* genes were found to be located on four distinct chromosomes, implying that the four *GhSPP* genes can be independently regulated for transcription in response to different internal signals or external environmental stresses.

To thoroughly analyze the structural characteristics and conservation of members within the *SPP* gene family of *hirsutum*, the MEME tool was employed to examine their conserved motifs. Six motifs were identified and integrated with gene structure (CDS) information for comprehensive analysis (Figure 3B). Research has found that the distribution of these motifs within genes exhibits certain patterns. Analysis results indicate that Motif 1 through Motif 6 are distributed across all four *GhSPP* genes, suggesting these motifs exhibit high conservation within the GhSPP family. Notably, the positions of Motif 5 and Motif 2 remain relatively stable in all four *GhSPP* genes, primarily located near the start region of the gene coding region. This suggests that these two motifs may be crucial for the core functions of GhSPP proteins. Genetic structure analysis indicates that the *SPP* gene in *hirsutum* exhibits a relatively complex structure, with its coding sequence (CDS) region interspersed by introns of varying lengths. The number of exons contained within each member shows significant variation: *GhSPP1*, *GhSPP2*, *GhSPP3*, and *GhSPP4* contain 16, 5, 8, and 8 exons, respectively. This constitutes a prominent feature of structural diversity among members of the *GhSPP* gene family.

### 3.4. Analysis of Cis-Transcription Elements on the Promoter of the G. hirsutum SPP Gene Family

**Figure 4:**
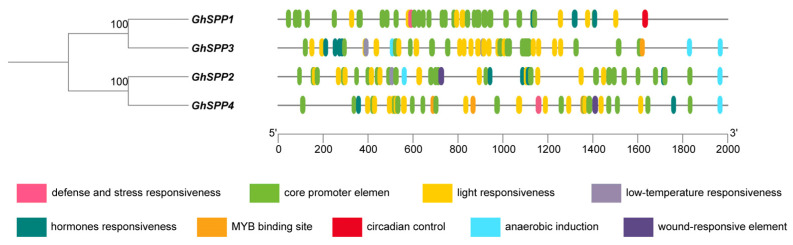
The cis-acting element of the *SPP* gene family of *hirsutum*.

To investigate the transcriptional regulatory mechanisms of the *SPP* gene family in *hirsutum*, this study selected sequences spanning 2000 bp upstream of the start codons of four *GhSPP* genes as promoter sequences for cis-acting element prediction analysis (Figure 4). Results indicate that the promoter regions of these genes are rich in diverse cis-acting elements with distinct functions, primarily categorized into three types: Light-responsive elements, hormone responsiveness elements (such as abscisic acid (ABA), salicylic acid (SA), and auxin response elements), and abiotic/biotic stress responsiveness elements (including defense and stress responsiveness, low-temperature responsiveness, and drought-inducible MYB binding sites). The widespread presence of these components suggests that the *GhSPP* gene may be broadly involved in *hirsutum*’s photoresponse, hormone signaling, and responses to abiotic stresses such as drought and low temperatures. Notably, the types and numbers of elements within the promoter regions of different *hirsutum* SPP family members show significant variation. Among them, *GhSPP3* contains the highest number of elements, while *GhSPP4* has the lowest, suggesting potential functional differentiation within the *GhSPP* gene family.

### 3.5. Genetic Collinearity Analysis of the G. hirsutum SPP Family

**Figure 5:**
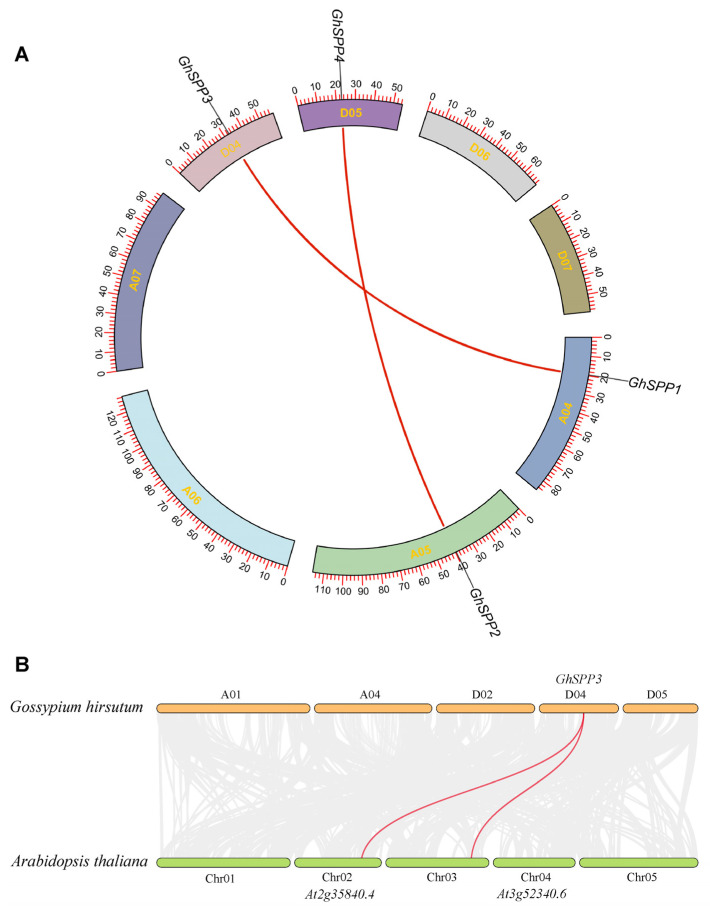
Genetic collinearity analysis of the GhSPP Family. (**A**) Collinearity analysis of *SPP* genes in *hirsutum*. (**B**) Collinearity analysis between *G. hirsutum* GhSPP genes and *Arabidopsis thaliana* genes.

To elucidate the evolutionary relationships among members of the terrestrial *hirsutum SPP* gene family, homology between *GhSPP* gene pairs was identified using the MCScanX software. The results revealed that the four *GhSPP* genes form two pairs of segmental duplication gene pairs, namely GhSPP1/GhSPP3 and GhSPP2/GhSPP4 (Figure 5A). By calculating the ratio of non-synonymous to synonymous substitution rates (Ka/Ks) for these homologous gene pairs, it was found that the Ka/Ks values for all gene pairs were less than 1 (Table 3), indicating that the evolution of the *GhSPP* gene family is primarily influenced by purifying selection. To further investigate the evolutionary relationships of *SPP* genes across different plant species, a cross-species synteny analysis was conducted between Arabidopsis thaliana and *hirsutum*. The results revealed (Figure 5B) two pairs of homologous *SPP* genes between the two species. This finding suggests that the *SPP* genes in *G. Hirsutum* and *A. thaliana* likely originated from a common ancestral gene and have maintained high conservation within dicotyledonous plants.

### 3.6. Analysis of Gene Expression Patterns in the G. hirsutum SPP Family

**Figure 6:**
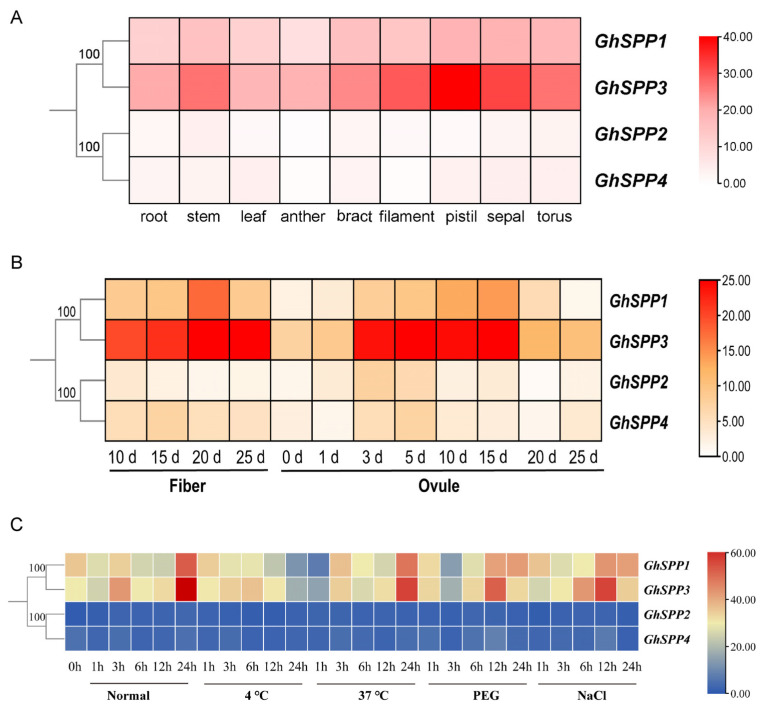
Analysis of gene expression patterns in the *GhSPP* Family. (**A**) Analysis of the expression patterns of the *GhSPP* gene in different tissues. (**B**) Expression patterns of the *GhSPP* gene in fiber and ovule at different post-flowering time points. (**C**) Analysis of the expression patterns of the *GhSPP* gene under different abiotic stresses.

To analyze the potential functions of *GhSPP* gene family members in the growth and development of *hirsutum*, this study analyzed the expression patterns of the four members *GhSPP1*-*GhSPP4* across nine tissue organs—roots, stems, leaves, anthers, bracts, filaments, pistils, sepals, and torus—based on transcriptome data, presenting the results as a heatmap (Figure 6A). Results indicate that all four *GhSPP* genes are expressed in the aforementioned tissues, though expression levels vary. Among them, *GhSPP1* and *GhSPP3* exhibit relatively high overall expression levels, with moderate-to-high-intensity expression signals detected in multiple tissues. The expression of *GhSPP1* exhibits significant tissue specificity, with its expression levels in anther tissue being markedly higher than in all other tissues tested. The tissue-specific high expression of *GhSPP1* in anthers suggests its potential function in male reproductive development, possibly playing a role in processes such as pollen formation. *GhSPP3* exhibits high expression levels across multiple tissues, with the highest expression in roots and filaments, followed by stems. This suggests its potential involvement in root physiology and the functional maintenance of specific floral organs such as filaments. Analysis of the expression profiles of the *SPP* gene family in fibers and ovules at different post-flowering time points is shown in Figure 6B. Among these, *GhSPP1* and *GhSPP3* maintained high expression levels at all detected time points, with further increases observed during the mid-to-late stages of fiber development (15–25 days). This suggests that *GhSPP1* and *GhSPP3* may play crucial roles in secondary wall thickening or cellulose synthesis in cotton fibers post-flowering. In contrast, *GhSPP2* and *GhSPP4* exhibited overall lower expression levels with relatively stable patterns across tissues and developmental stages, suggesting they may perform basal metabolic or constitutive functions. Furthermore, distinct expression patterns were observed for the same gene between fibers and ovules. For example, *GhSPP3* exhibited strong expression during early ovule development (3–15 days), whereas its peak expression in fibers occurred later (15–25 days), indicating potential stage-specific regulation of this gene across different tissues.

To investigate the role of the *GhSPP* gene in abiotic stress responses, this study analyzed the expression patterns of *GhSPP1–GhSPP4* under low-temperature (4 °C), heat shock (37 °C), PEG-simulated drought, and NaCl salt stress treatments (Figure 6C). The results indicate that there are significant differences in how members respond to coercion. *GhSPP1* and *GhSPP3* exhibit rapid and sustained strong induction of expression during osmotic stress (PEG and NaCl) treatment, suggesting they may function in responding to drought and salt stress. Among these, *GhSPP3* showed the most significant induction of expression, particularly under salt stress, where its expression level consistently remained the highest among all family members, indicating that *GhSPP3* is a core member of the *hirsutum SPP* gene family that is highly responsive to abiotic stress and is likely play a key role in the mechanisms of salt tolerance and drought resistance in cotton. Additionally, *GhSPP1* also exhibited an upregulation trend under heat stress treatment, suggesting that it may also participate in the heat stress response. In contrast, the expression pattern of *GhSPP2* exhibits high stress specificity and temporal delay, with its expression significantly upregulated only 24 h after cold treatment, suggesting it may specifically participate in *hirsutum*’s cold adaptation pathway. *GhSPP4*, however, shows relatively weak expression under all stress conditions, indicating it likely performs fundamental constitutive functions or plays a supplementary regulatory role in stress responses. These differential expression patterns, combined with the abundance of stress-responsive cis-acting elements within their promoters (Figure 4), collectively indicate that the *GhSPP* gene family possesses significant and distinct regulatory potential in land cotton’s adaptation to complex abiotic stress environments. This provides crucial clues for elucidating their specific molecular mechanisms of stress resistance in subsequent studies.

### 3.7. Genetic Protein Interaction Analysis of the G. hirsutum SPP Family

**Figure 7:**
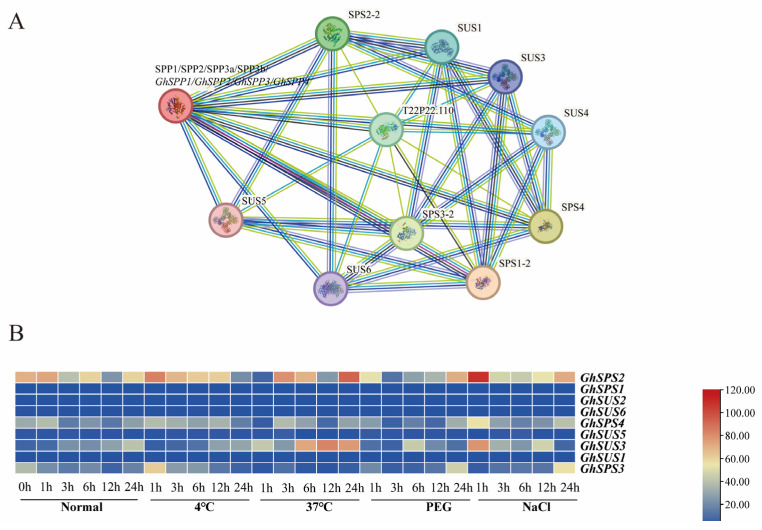
Genetic protein interaction analysis of the GhSPP family. (**A**) GhSPP interaction protein network. (**B**) Regulating the expression patterns of genes encoding different interacting proteins under abiotic stress.

To investigate how the *SPP* gene functions in *hirsutum*’s resistance to abiotic stress, protein interaction network predictions indicate that the *GhSPP* gene family interacts with genes involved in sucrose synthesis (the *SUS* and *SPS* gene families) (Figure 7A). Analysis of the expression levels of *SUS* and *SPS* gene family members in *hirsutum* under abiotic stress revealed that *SPS2*, *SPS3*, *SPS4*, and *SUS3* genes were significantly upregulated under salt and drought stress (Figure 7B), with *SPS2* exhibiting the highest expression levels under both stresses. Based on our protein interaction predictions and co-expression patterns, we hypothesize that *hirsutum* may achieve efficient compartmentalization of sucrose synthesis in compartments such as chloroplasts by forming a “SPS-SPP-SUS” integrated synergistic metabolic regulation pathway. This hypothesis warrants further experimental validation.

## 4. Discussion

SPP specifically catalyzes the hydrolysis of sucrose-6-phosphate to sucrose, playing a crucial role in plant sugar metabolism, yield formation, and responses to abiotic stress. The *SPP* gene family is a small gene family with four members in *Arabidopsis*: *AtSPP1, AtSPP2*, *AtSPP3a*, and *AtSPP3b* [23,24,25,26,27]. In crops such as *Z. mays*, *O. sativa*, *S. bicolor*, *Triticum aestivum*, *Hordeum vulgare*, *Solanum tuberosum*, and *S. lycopersicum*, it contains 1 to 4 members. This study conducted a genome-wide identification and systematic analysis of the *GhSPP* gene family in *hirsutum*, identifying four members (*GhSPP1*–*GhSPP4*). Their number is comparable to that of SPP genes in other plants. This lays the foundation for further elucidating the functional roles of this family in *hirsutum*.

The physicochemical analysis results indicate that members of this gene family share similar physicochemical properties such as molecular weight. However, *GhSPP1* stands out significantly in terms of sequence length, molecular weight, and tissue expression levels, suggesting that it may have undergone functional diversification following gene duplication to fulfill specific biological roles. Subcellular localization predictions indicate that GhSPP3 and GhSPP4 localize to the cytoplasm, Consistent with the cytoplasmic localization of SPP proteins in most reported plants, including *Arabidopsis*, *Z. mays*, and *Beta vulgaris* [28]. GhSPP1 and GhSPP2, however, are predicted to be localized to the chloroplast, a finding that differs from previous studies. This finding strongly suggests that in *hirsutum*, at least part of the final steps of sucrose synthesis—particularly the SPP-catalyzed hydrolysis of Suc6P—may occur within chloroplasts. This represents a potential manifestation of species-specific functional regulation in *hirsutum*. Consequently, the functional patterns of *hirsutum* SPP family members in regulating sucrose synthesis differ from those observed in other plants.

Phylogenetic tree analysis grouped 20 *SPP* genes from seven plant species into five evolutionary clusters. Groups II and III predominantly comprised monocotyledons (*O. sativa*, *Z. mays*, and *S. bicolor*), while Groups I, IV, and V mainly consisted of dicotyledons (*G. hirsutum*, *S. lycopersicum*, *Arabidopsis*, and *S. lycopersicum*). These results support the notion that the *SPP* gene family underwent expansion following the monocotyledonous–dicotyledonous divergence within angiosperms. Analysis of conserved motifs further corroborates the reliability of the phylogenetic tree constructed in this study. Analysis of cis-acting elements in the promoter revealed that the *GhSPP* gene promoter region contains multiple abiotic stress response elements. This finding is consistent with the characteristic of *SPP* gene promoters in species such as *Arabidopsis*, *C. quinoa*, and *O. sativa*, which are also enriched with numerous cis-regulatory elements associated with stress responses. Furthermore, the *GhSPP* gene promoter exhibits the highest number of light-responsive cis-acting elements among *G. hirsutum SPP* genes, indicating that *GhSPP* gene expression is strongly regulated by light signals.

Analysis of the expression profiles of the *GhSPP* gene family across different tissues revealed that *GhSPP1* and *GhSPP3* are expressed in all tissues, with higher expression levels in flowers, sepals, and stems compared to other parts. This may be related to the sucrose supply required for *hirsutum* fiber development. This expression pattern shares similarities with other species while also exhibiting differences. For example, the *Arabidopsis AtSPP* gene also shows high expression in floral organs, whereas the maize *ZmSPP* gene is highly expressed in roots. The economic yield of cotton is concentrated in the cotton fiber (seed trichomes) [29], whose development depends on sucrose supply from floral organs (ovules) [30]. The significant increase in *GhSPP1* expression during the late stage of fiber development suggests its specific expression during this phase (typically corresponding to secondary wall thickening and peak cellulose synthesis) to support the substantial sucrose supply required for the biosynthesis of fiber cell wall components such as cellulose. *GhSPP3* exhibits widespread high expression in multiple reproductive organs including pistil, filament, and sepal, while maintaining consistently high expression throughout fiber and ovule development. Its overall gene expression level is markedly higher than other family members, suggesting *GhSPP3* is likely the primary contributor to sucrose synthesis in cotton. Its high expression in reproductive organs suggests involvement in anther development, pollen formation, pollen wall synthesis, and filament elongation, providing the fundamental carbon flux for these processes. This elevated expression ensures the plant produces healthy, viable pollen for successful pollination. Its dominant expression during ovule development likely provides immediate energy and carbon skeletons for rapid post-fertilization ovule division, differentiation, and protrusion of primary fiber cells, establishing it as an early foundation gene for fiber yield formation. Its sustained high expression during fiber development may collaborate with *GhSPP1* to support continuous fiber elongation and thickening.

Analysis of *GhSPP* gene family members under abiotic stress treatments revealed that *GhSPP1* and *GhSPP3* showed significantly upregulated expression under salt and drought stress. This finding strongly aligns with previous observations that the promoters are rich in abiotic stress response elements (such as ABRE, DRE, etc.). Notably, the upregulation of *SPP* genes under salt and drought stress has been reported in various plants including *Arabidopsis*, *C. quinoa*, and *O. sativa*, suggesting that the function of *SPP* in plant stress responses may exhibit a degree of conservation. Furthermore, the expression levels of *GhSPP3* under drought and salt stress were significantly higher than those of other family members, indicating that *GhSPP3* is the core member of the *GhSPP* gene family in responding to abiotic stress.

Predictions from the protein interaction network reveal a synergistic regulatory mechanism for key sucrose metabolism genes in *G. hirsutum* under abiotic stress. Based on these findings, we hypothesize that the interaction between *SPS* and *SPP* forms an “SPS-SPP complex,” enabling substrate channeling—enabling direct transfer of the intermediate product sucrose-6-phosphate and avoiding diffusion losses within the cell. This facilitates efficient, directed sucrose synthesis under stress conditions, providing an energy source for plants to resist abiotic stress and enhance their resilience.

In summary, the *GhSPP* gene family exhibits functional differentiation in protein structure, evolutionary relationships, and expression patterns, with *GhSPP3* potentially playing a central role in abiotic stress responses. Compared to plants such as *Arabidopsis* and *Z. mays*, the *SPP* gene family in upland cotton exhibits species-specific characteristics, such as subcellular localization predictions, while also demonstrating functional conservation in aspects like phylogenetic evolution and stress response expression. Subsequent studies will validate its biological functions and regulatory mechanisms through overexpression, gene knockout, and chromatin immunoprecipitation experiments. These findings will be subjected to in-depth comparative analysis with results from other species to comprehensively elucidate the unique role and shared principles of *SPP* in cotton, a major cash crop.

## Figures and Tables

**Table 1:** Physicochemical properties of the *SPP* gene family of *G. hirsutum*.

	GhSPP1	GhSPP2	GhSPP3	GhSPP4
Gene ID	Ghi_A04G02881	Ghi_A05G16746	Ghi_D04G05296	Ghi_D05G10991
Length (aa)	1015	335	427	456
MW (kDa)	113.28	38.38	48.12	52.17
pI	5.39	5.87	5.99	6.33
Instability index	39.28	40.81	35.03	38.68
Subcellular location	chloroplast	chloroplast	cytoplasm	cytoplasm

**Table 2:** Secondary Structure of *G. hirsutum* SPP Protein.

Protein	Alpha Helix	Extended Strand	Beta Turn	Random Coil
GhSPP1	41.18%	13.79%	7.09%	37.93%
GhSPP2	43.28%	15.22%	4.18%	37.31%
GhSPP3	40.05%	15.22%	4.92%	39.81%
GhSPP4	40.35%	14.91%	4.82%	39.91%

**Table 3:** Ka/Ks ratio for *SPP* colinear gene pairs in *G. hirsutum*.

Duplicated Gene Pairs	Ka	Ks	Ka/Ks
*GhSPP1*/*GhSPP3*	0.00917	0.02776	0.33033
*GhSPP2*/*GhSPP4*	0.04326	0.09042	0.47843

## Data Availability

The original contributions presented in the study are included in the article, further inquiries can be directed to the corresponding authors.
